# Environmental Engineering Applications of Electronic Nose Systems Based on MOX Gas Sensors

**DOI:** 10.3390/s23125716

**Published:** 2023-06-19

**Authors:** Ali Khorramifar, Hamed Karami, Larisa Lvova, Alireza Kolouri, Ewa Łazuka, Magdalena Piłat-Rożek, Grzegorz Łagód, Jose Ramos, Jesús Lozano, Mohammad Kaveh, Yousef Darvishi

**Affiliations:** 1 Department of Biosystems Engineering, University of Mohaghegh Ardabili, Ardabil 56199, Iran; a.khorramifar@uma.ac.ir (A.K.); alirezakolouri@uma.ac.ir (A.K.); 2 Department of Petroleum Engineering, Knowledge University, Erbil 44001, Iraq; sirwan.kaweh@knu.edu.iq; 3 Department of Chemical Science and Technologies, University of Rome “Tor Vergata”, 00133 Rome, Italy; 4 Department of Applied Mathematics, Faculty of Technology Fundamentals, Lublin University of Technology, 20-618 Lublin, Poland; e.lazuka@pollub.pl (E.Ł.); m.pilat-rozek@pollub.pl (M.P.-R.); 5 Department of Water Supply and Wastewater Disposal, Faculty of Environmental Engineering, Lublin University of Technology, 20-618 Lublin, Poland; g.lagod@pollub.pl; 6 College of Computing and Engineering, Nova Southeastern University (NSU), 3301 College Avenue, Fort Lauderdale, FL 33314-7796, USA; jr1284@nova.edu; 7 Department of Electric Technology, Electronics and Automation, University of Extremadura, Avda. De Elvas S/n, 06006 Badajoz, Spain; jesuslozano@unex.es; 8 Department of Biosystems Engineering, University of Tehran, Tehran P.O. Box 113654117, Iran; sdarvishi@ut.ac.ir

**Keywords:** e-nose, olfactory system, metal oxide semiconductor sensors, environment contamination monitoring, olfactory classes, volatile compounds, air, specific applications, water and wastewater management

## Abstract

Nowadays, the electronic nose (e-nose) has gained a huge amount of attention due to its ability to detect and differentiate mixtures of various gases and odors using a limited number of sensors. Its applications in the environmental fields include analysis of the parameters for environmental control, process control, and confirming the efficiency of the odor-control systems. The e-nose has been developed by mimicking the olfactory system of mammals. This paper investigates e-noses and their sensors for the detection of environmental contaminants. Among different types of gas chemical sensors, metal oxide semiconductor sensors (MOXs) can be used for the detection of volatile compounds in air at ppm and sub-ppm levels. In this regard, the advantages and disadvantages of MOX sensors and the solutions to solve the problems arising upon these sensors’ applications are addressed, and the research works in the field of environmental contamination monitoring are overviewed. These studies have revealed the suitability of e-noses for most of the reported applications, especially when the tools were specifically developed for that application, e.g., in the facilities of water and wastewater management systems. As a general rule, the literature review discusses the aspects related to various applications as well as the development of effective solutions. However, the main limitation in the expansion of the use of e-noses as an environmental monitoring tool is their complexity and lack of specific standards, which can be corrected through appropriate data processing methods applications.

## 1. Introduction

**Figure 1:**
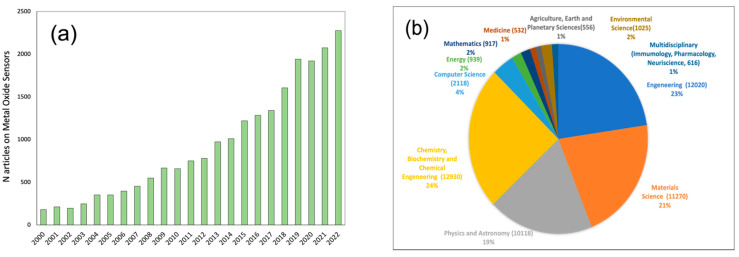
The diagrams of (**a**) over year progress on MOXs sensors publications; (**b**) statistics on MOX sensors application fields. Citation report created from Scopus by searching as topic “e-nose” or “electronic nose” as well as referring to the previous search with the sub-topic “metal” and “oxide”. Search limited to the document types: article, review, conference paper, book, book chapter. Last accessed 6 April 2023.

E-noses, through mimicking the olfactory system of humans, can detect various smells, which makes them suitable for an extensive range of environmental analysis fields, applications in the food industry, medical practice for disease diagnosis, etc. [1,2,3,4,5]. Among the different types of chemical sensors employed in e-nose technology, the metal oxide semiconductor sensors (MOXs) are the most actively used class. According to the data from the Scopus database (statistic from 17 March 2023), among the 3023 e-nose systems reported from 1974 up to nowadays, more than 35% (1073 sensing systems) were based on MOX sensors’ technology [6]. Thus, the analysis of the last 20 years of publications on MOXs indicates first of all the continuous growth of the scientists’ interest in the development of these types of sensing devices: from 180 papers published in 2000 to 2276 research works in 2022 and 561 papers already published in first trimester of 2023; see Figure 1a. Among this research, the chemical, biochemical and chemical engineering applications of MOXs sensors account more than 24% of all research studies in the field, which are followed by engineering (23%), material science (21%), physics and astronomy (19%), computer science (4%), energy (2%), medicine (1%), and other multidisciplinary applications; see Figure 1b.

**Figure 2:**
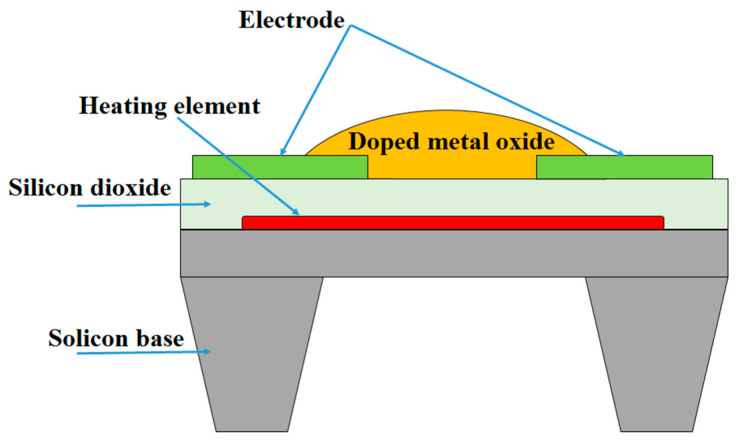
The schematic presentation of MOX sensor based on information from [9,10].

Typically, MOX sensors are resistive sensors made of metal oxide semiconductors which have been regarded as a suitable candidate for the development of low-cost and highly efficient sensors due to their simple physical basis in the measurement of the resistance as well as high sensitivity to different gases [7]. The MOX sensors are also called chemical resistance or semiconductor sensors. These devices are often used to evaluate volatile compounds at ppm and sub-ppm levels, and compared to amperometric gas sensors (AGSs, i.e., electrochemical gas sensors based on amperometry), MOXs are one of the promising technologies for the detection of Inorganic gases at low concentration levels. They can be also utilized as an alternative technology for the assessment of some AGS-available gases such as carbon monoxide [8]. The schematic presentation of an MOX sensor is given in Figure 2. The working principle of the MOX device is based on the changes in the electrical resistance of the specific metal oxide semiconductor (for instance SnO_2_) upon exposure to gases at high temperatures (150–500 °C). The sensing material is placed on a substrate with integrated electrodes (to retrieve the electrical resistance) and a vapor resistor (to heat up the sensing materials).

The MOX sensors were first introduced as resistive chemical gas sensors in 1952 by Brattain and Bardeen, who showed that some semiconductor materials change their resistance depending on the surrounding atmosphere [11]. Conductivity variations occur in MOXs due to electron transmission with the adsorption and desorption of gas molecules, in which the shape and size of nanostructures play an important role. Material properties are also essential for increasing the sensitivity of sensors (such as electronic, morphological, and chemical properties, catalyst dispersion, Fermi surface position, crystal size, and their network connection) [12]. Among them, tin oxide is one of the most frequently used thanks to its high sensitivity, low temperature, and low fabrication costs compared to other semiconductors [13].

Although MOX sensors are intrinsically non-specific, their sensitivity can be improved by some techniques, among which multivariate predictive models [14], application of the dynamic response of a transient sampling or temperature modulation [15,16], doping the metal oxide layer with noble metals [17], and the use of chemical filters [18,19] are more popular.

The MOX sensors are smaller, faster (response time of 10 to 20 s), and more durable than the above-mentioned AGS sensors; however, MOXs suffer from susceptibility to humidity, which can be resolved by modulation of the working temperature [20]. Thanks to the microelectromechanical systems (MEMS), MOX sensors can be deposited on a miniature layer on a small thermal plate, creating a footprint of few mm^2^, a response time of 5–10 s, and a power rating of 15–30 mW. The response time and energy consumption rate can be decreased by signal processing techniques [21,22] as well as acquisition cycles [22]. Additionally, the sensor housing (which is essential for the placement of all sensors) increases the response time; on the other hand, the use of electricity might be problematic. Therefore, designing small housings is essential for fast measurements [21].

Controlling and monitoring the emission of combustion-related greenhouse gases has become a top priority of many industrial processes for the reduction in energy consumption, improvement of product quality, and protection of the environment [23,24]. Therefore, sensor-based smart systems have been widely used in various fields, especially in the detection of toxic and flammable gases. Energy efficiency and other industrial and environmental applications require the development of fast, reliable, sensitive, selective, and low-cost sensors. Industrial processes have long relied on gas detection systems using infrared spectroscopy, gas chromatography/mass spectrometry (GC/MS), and also chemiluminescence with limitations in proper detection and fast response times [25]. These instruments are expensive, bulky, incompatible with high-temperature environments, and they require maintenance and gas sampling systems [26,27]. Therefore, small and stable gas sensors capable of detecting odors over a wider range at high sensitivity and selectivity are essential for online monitoring at low and high temperatures with short response and recovery times [28].

The low power requirement, simplicity, lightweight, the possibility of merging with fixed measurement systems, and portability are among the major advantages of MOX sensors [29]. Thus, for instance, these sensors are the basis of industrial and domestic carbon monoxide alarms [30], and they also have been employed in the e-noses systems for various applications including medicine [31], pharmacy [32], food quality control [33,34,35,36,37,38,39,40], agriculture [33,41,42,43], and odor classification [42,43,44,45,46], among others.

This article investigates the MOX sensors and e-nose sensor arrays applications in the environmental monitoring and presents the achievements in the field over the last 5 years. The review includes several sections dedicated to the description of different principles of operation of metal oxides as gas sensing materials, which are followed by the description of the selected applications of different types of MOXs for evaluation of the pollutant gases, air quality assessment, and water and wastewater pollution evaluation.

## 2. Principle of Operation of MOX Sensors

Work on MOX semiconductor metal oxide sensors has been ongoing since the mid-20th century. The gas-sensitive properties of germanium oxide were described in 1953 [11]. The first gas sensors were made of zinc oxide ZnO, which was followed by the use of such materials as SnO_2_, TiO_2_, WO_3_, Ga_2_O_3_, In_2_O_3_ or Fe_2_O_3_. Out of the aforementioned materials, granular tin dioxide is the most widely studied and used; it has a developed surface on which the adsorption/desorption phenomenon of the studied gases occurs [47,48]. Depending on the technique for producing the gas-sensitive layer, the sensors are divided into thin-film sensors with a layer thickness of 5 nm to 2 µm and thick-film sensors with a layer thickness of 10–300 µm. Sputtering or evaporation techniques are used to deposit thin-film gas-sensitive material. In thick-film ones, a layer of paste containing metal oxides is applied and then fired at high temperature. Thick-film sensors, compared to thin-film ones, are less susceptible to poisoning, are more durable, and exhibit higher sensitivity [48,49].

**Figure 3:**
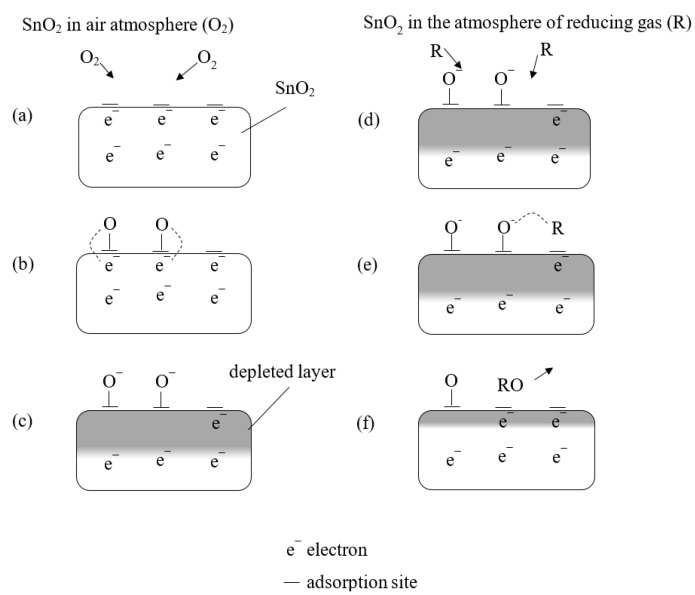
Conductance change mechanism of a semiconductor as a result of ion exchange in the atmosphere of air and reducing gas, where: (**a**) oxygen binding from the air, (**b**) oxygen sharing electrons from the semiconductor, (**c**) formation of a surface layer depleted of electrons, (**d**) binding of gas molecules to oxygen atoms, (**e**) return of electrons to the semiconductor, (**f**) reduction of the surface electron-depleted layer [48,51,52].

MOX sensors allow measuring gas concentrations through the phenomenon of changing the electrical conductivity of a semiconductor receptor–transducer element. In MOX sensors, the measuring element is usually a tin dioxide sinter. Depending on the purpose of the sensor, the semiconductor sinter contains various dopants in the form of precious metals. The change in electrical conductivity in the semiconductor layer is the result of a chemisorption process, i.e., the formation of chemical bonds between gas molecules and the semiconductor. This leads to a change in the concentration of current carriers on the surface of the semiconductor and thus a change in electrical conductivity [47,50]. The mechanism of signal formation in a semiconductor is shown in Figure 3. Adsorption sites in oxygen vacancies of the surface layer of semiconductor grains are of fundamental importance. Vacancies are point defects in the crystal lattice consisting of the non-occupancy of lattice nodes with atoms or ions. They constitute active centers, i.e., fragments of the receptor–transformer element involved in oxygen chemisorption. In the vacancies, oxygen is bound from the air (Figure 3a), which shares electrons from the semiconductor (Figure 3b). As a result, a surface layer of weak conductance is formed in the semiconductor, which is depleted of electrons (Figure 3c). Depending on the oxygen partial pressure and temperature, an equilibrium state is produced in the system for the vacancy concentration and oxygen concentration. During exposure of the semiconductor to the reducing gas, the reaction is reversed. Gas molecules bind to oxygen atoms (Figure 3d). The electrons shared by oxygen return to the semiconductor (Figure 3e), reducing the surface electron-depleted layer, a symptom of which is an increase in conductance (Figure 3f). The reaction of the reducing gas with the adsorbed oxygen produces molecules of chemical compounds such as CO, CO_2_ or H_2_O [48,51].

**Figure 4:**
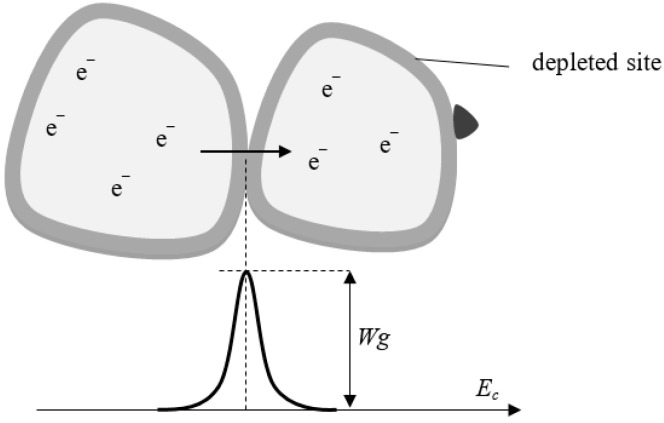
Lower limit of the conduction band *E_c_* (conduction band model) and the value of the energy barrier *W_g_* [53,54,55].

An energy barrier *W_g_* is formed between the grains of the semiconductor, inhibiting the flow of electrons. The energy barrier is a range of energy equal to the work of exit of electrons from a semiconductor grain, which is defined in the electron volt unit. When a semiconductor is in a pure air atmosphere, the depleted layer increases, and thus, the energy barrier between grains is increased as well (Figure 4). The energy barrier is lower when the semiconductor is in an atmosphere of reducing gaseous impurities [12]. From a practical point of view, this means that the resistance R decreases when the sensor is in an atmosphere of polluted air. The resistance of the sensor can be expressed by the formula [47,53]:

(1) $$R=R_{Z}\exp\left(\frac{W_{g}}{k_{B}T}\right),$$

where $R_{z}$—internal grain resistance (Ω), $W_{g}$—energy barrier (eV), *k_B_*—Boltzmann constant (eV·K^−1^), *T*—temperature (K).

Depending on the temperature of the semiconductor substrate, different patterns of oxygen binding on the semiconductor layer can be distinguished. At low temperature, weak physical adsorption by van der Waals forces dominates, with a binding strength of less than 0.1 eV. Between the adsorbed gas molecules and the semiconductor surface, there is no exchange of electric charge and the resulting change in the concentration of carriers in the gas-sensitive layer, so low-temperature adsorption does not cause a change in conductivity [56]. Below 400 K, O_2_^−^ ionic forms appear, while at temperatures above 400 K, the more stable adsorbed O^−^ and O_2_^−^ electron-rich ionic forms of oxygen begin to appear, which are responsible for the useful properties of oxide sensors [12]. This results in the manufacturers’ recommended operating temperature of the receptor–transducer element, in which oxygen ionic forms are dominant at the range of 630–650 K. The adsorbed oxygen ionic forms create strong covalent bonds with electrons from the semiconductor surface layer with a binding energy of 0.6 to 1 eV between the gas molecules and the semiconductor surface. Increasing the temperature above 700 K leads to the formation of surface and volume defects in the semiconductor.

Sensitivity and selectivity are important parameters in the selection of sensors for the array. Improvement of these sensor parameters is performed by [47,48]:

- Development of new gas-sensitive materials;
- Modification of the sensor design;
- Uniform temperature distribution of the receptor element;
- Use of doping substances;
- Incorporation of a catalytic filter.

**Figure 5:**
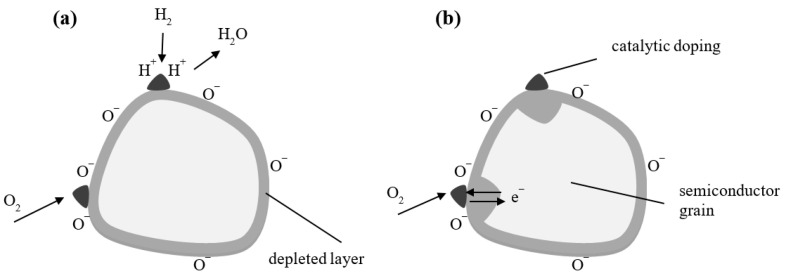
Mechanisms of action of catalytic dopants: (**a**) chemical model, (**b**) electrical model [48,61].

In the case of SnO_2_, the best sensitivity is obtained when the effective Debey length of the LD is close to the grain radii of the semiconductor; for this reason, the use of polycrystalline and nanocrystalline structures is preferred. The use of catalytic dopants in the gas-sensitive material has a very good effect on improving selectivity. The most common are precious metals such as gold, palladium, platinum and silver [57,58,59]. The mechanism for improving sensor sensitivity with dopants is described by two models: chemical and electrical. The chemical model assumes that there is increased oxygen or hydrogen bonding on the surface of catalytic dopants (Figure 5a). The catalyst increases the number of dissociated molecules while decreasing the energy required to dissociate the gas molecules. The products of dissociation are weakly bound to the catalyst and are displaced to the grain surface. In the electrical model (Figure 5b), the grain conductance is affected by the contact between the catalyst and the grain surface. The partially oxidized metallic catalyst binds electrons from the semiconductor so that the width of the depleted layer increases [48,60,61].

Knowing the resistance of the sensor in the gas atmosphere, the gas concentrations can be read using the characteristics provided by the manufacturer. In oxide sensors, a thin layer of SnO_2_ paste is applied to a ceramic substrate and then fired at 970–1270 K. The substrate is a tube with an inner diameter of 1 mm or a plate with good thermal conductivity (20 W/m·K), made of Al_2_O_3_. A heating coil with a resistance of a few dozen ohms is placed inside the tube or on the bottom layer of the plate. Metallic electrodes (Au or Pt) are sputtered at the ends of the ceramic tube to allow electrical connection of the semiconductor [48,50].

Gas sensors operate in a variety of environmental conditions and atmospheres of varying composition. Changes in sensor characteristics can also occur due to [47,50]:

- Occupancy of active centers by interfering compounds;
- The co-reaction of gaseous pollutants;
- The presence of water vapor.

Gas sensors operating in environments with high concentrations of chemical compounds are particularly prone to permanent poisoning, which is associated with irreversible changes in their characteristics. This phenomenon necessitates cyclic calibration. The studies conducted by Romain et al. [62] on the long-term stability of MOX sensors showed that there was a significant change in sensor characteristics after 7 years. Longer life can be provided by reducing the sensor’s exposure time to contaminants. Sequential sampling for a short time of a gas sample combined with flushing with clean air is a good solution.

## 3. Semiconductor Metal Oxides as Gas-Sensing Materials

Metal oxides, MOXs, are widely employed in modern sensing materials due to their tunable physical and chemical properties. The performance of the MOXs depends on their chemical and structural features including the chemical composition, morphology, structural defects, specific surface area, grain size, etc. [63]. The variation of any of these features could provide control over the sensor features. Thanks to their unique properties, MOXs are among the most diverse classes of materials covering almost all aspects of material science and physics in the fields of superconductivity, semiconductors, magnetic, and ferroelectric materials [64]. Several MOXs, such as for instance SnO_2_, In_2_O_3_, ZnO, WO_3_, CdO, and TiO_2_, are used as the sensing material in the semiconductor sensors. These distinct transparent conductive oxides are characterized by sufficient electrical conductivity, high reactivity, and a wide spectrum of transparency. Among the above-mentioned MOXs, TiO_2_ and SnO_2_ are the two most popular due to their low fabrication cost, mechanical and chemical stability, and high thermal resistance and adhesion to glass [28]. The selection of metal oxide materials for gas-sensing applications depends on factors such as their electrical conductivity, reactivity, stability, sensitivity to specific gases, and cost. The specific requirements of each application, such as the target gases to be detected, operating conditions (temperature, humidity), and desired sensing performance, also play a crucial role in determining the choice of metal oxide materials.

The more detailed description of TiO_2_ and SnO_2_ properties, including the possible sensing material modifications, as well as the main characteristics, sensing properties and gas sensing applications of common MOXs materials will be provided in the following sections of this review.

### 3.1. Titanium Oxide (TiO_2_)

Titanium dioxide (TiO_2_) is a widespread, non-toxic, inexpensive, and environmentally friendly compound [65] that makes it suitable for various applications in biosensors, UV sensors, photocatalysts, humidity sensors, CO_2_ reduction and H_2_ production processes. Being an n-type semiconductor, TiO_2_ is widely used in the development of conductometric gas sensors [66]. As a sensing material, TiO_2_ benefits from large and reversible resistance variations along with exceptional high-temperature chemical stability; it can be found in three major phases: brookite (orthorhombic), anatase (tetragonal), and rutile (tetragonal) phases with energy bandgaps of 3 eV (rutile), 3.2 eV (anatase) and 3.13–3.40 eV (brookite), respectively [67]. Among these crystal phases, the rutile is the constant and major phase, while the anatase phase offers better outcomes in the gas-sensing applications. For instance, anatase gas sensors are employed in solar cells (due to their intrinsic properties such as high electron mobility, low density, and small dielectric constant) [68]. TiO_2_ is used as the photoactive layer in the gas sensor in which the mechanism of chemical resistance and conductivity is based on the adsorption process or repulsion of reducing and oxidizing gases [69]. Some parameters of TiO_2_ nanostructures should be improved to further extend their applications: for instance, sensor signal, conductivity of TiO_2_ in the air, response and recovery level as well as doping [28]. TiO_2_ gas sensors find applications in biosensing, UV sensing, photocatalysis, and humidity sensing. Tin oxides (SnO and SnO_2_) gas sensors are utilized for carbon monoxide detection, environmental monitoring, industrial processes, and breath analysis. Some of the industrial processes where tin oxide gas sensors find application include the following. First, they are useful in combustion monitoring. Tin oxide gas sensors can be used in industrial combustion processes, such as those in power plants or manufacturing facilities, to detect the presence of carbon monoxide (CO) and ensure efficient and safe combustion. Second, there is emissions control. In industries that produce harmful gases as by-products, such as automotive manufacturing or chemical plants, tin oxide gas sensors can be utilized to monitor and control emissions, ensuring compliance with environmental regulations. Third, they are of use in the petrochemical industry. Tin oxide gas sensors play a vital role in the petrochemical industry, where they are used to detect and monitor volatile organic compounds (VOCs) and other gases that may be present in the production processes or storage facilities. A fourth area is semiconductor manufacturing. Tin oxide gas sensors find application in the semiconductor industry, where they are used to monitor and control gas concentrations during the manufacturing processes to ensure the quality and safety of semiconductor devices. Lastly, there are applications in the food and beverage industry. In the food and beverage industry, tin oxide gas sensors can be utilized for gas monitoring in storage facilities, fermentation processes, and packaging environments, helping to maintain product quality and safety.

### 3.2. Tin Oxides (SnO and SnO_2_)

Tin oxide (SnO) is a p-type semiconductor with a direct bandgap of 2.5–3.0 eV [70]. Tin oxide (SnO_2_), also called cassiterite, has a rutile structure similar to other metal oxide semiconductors. Compared to SnO_2_, SnO is unstable at temperatures above 270 °C. In general, the gas sensors are made of thick porous SnO_2_ film with a higher surface-to-volume ratio. When the material is heated up, the electrons are entrapped and adsorbed by the molecules of material; moreover, the energy band bending alters the conductivity [71].

SnO_2_, due to its low-temperature reactivity (which varies from room temperature to several hundreds of degrees Celsius depending on the gaseous analyte type), has been widely used in a variety of applications, including biosensors, humidity sensors, UV sensors, photocatalysts, batteries, and thin film transistors. SnO_2_ belongs to the group of surface-sensitive materials [72]. Changes in the electrical conductivity of sensor materials can be attributed to conduction band diffusion, and changes in the charge carrier concentration are due to high charge mobility.

For SnO_2_, the chemisorbed oxygen (or adsorbed ion) and other molecules with net electric charge are the main stimuli of response, rather than the oxygen composing SnO_2_ lattice. The presence of these charged species can decrease or increase the SnO_2_ surface conductivity, and, as a consequence, vary the gas response signal intensity. Moreover, SnO_2_ performance can be modified by additives in order to increase the charge carrier concentration by donor atoms or enhance the gas sensitivity or catalytic activities by metallic additives [28]. Among other MOXs, SnO_2_ is the best material for CO gas sensing [71]. Tin oxide (SnO and SnO_2_) gas sensors find applications in various industrial processes, environmental monitoring, and breath analysis. In industrial processes, they are used for combustion monitoring, ensuring efficient and safe combustion in power plants and manufacturing facilities. They also play a role in emissions control, helping to monitor and regulate the release of harmful gases in industries such as automotive manufacturing and chemical plants. They contribute to assessing and improving overall air quality in various settings, including urban areas, industrial zones, and residential environments. Furthermore, tin oxide gas sensors are employed in breath analysis applications. They can detect and measure certain gases present in human breath, providing valuable insights for medical diagnostics and the monitoring of respiratory conditions.

### 3.3. Zinc Oxide (ZnO)

**Figure 6:**
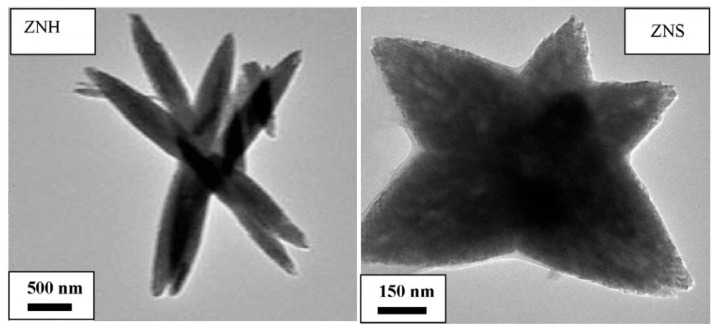
Shape/morphology of different samples of Zinc oxide.

Zinc oxide (ZnO) is a cost-effective, non-toxic, abundant, and chemically stable n-type semiconductor (due to local defects such as Zn interstitials and O vacancies) with a wide energy bandgap and large excitation binding energy [73]. Polar surfaces have different physical and chemical properties compared to non-polar ones, and O-polar surfaces also have different electronic structures. These features play a vital role in the development of ZnO properties such as crystal growth, polarization, and defect. For gas-sensing purposes, the polar surface of Zn is more active than the O-polar and non-polar surfaces (due to the formation of active OH^−^ ions) [74]. ZnO can be also employed in other applications, such as solar cells, humidity sensors, photocatalysis, UV sensors, biosensors, and field-effect transistor sensors [74]. According to the Krishnakumar and coworkers, various morphologies of ZnO can be attained by changing synthesis parameters such as precursors and microwave irradiation time [75]. The shape/morphology of different samples of ZNA (zinc acetate, liquid ammonia, pH8 (Natural Alkaline Water)), ZNH (zinc nitrate, hydrazine hydrate) and ZNS (zinc nitrate, PVP, liquid ammonia) are spherical, flower, and star, respectively, as depicted in the following Figure 6 [75]. Zinc oxide (ZnO) gas sensors are used for gas detection, environmental monitoring, industrial processes, and breath analysis applications. In the context of zinc oxide (ZnO) gas sensors, environmental monitoring and industrial processes encompass a range of applications where these sensors are utilized for gas detection and analysis. For one, there is the environmental monitoring of indoor air quality. ZnO gas sensors are used to monitor and assess the quality of indoor air in various settings such as homes, offices, schools, and public buildings. They can detect gases released from building materials, furniture, cleaning products, and other indoor pollution sources.

In addition, they are also useful in industrial processes and maintaining industrial hygiene. ZnO gas sensors are used in industrial settings to monitor workplace air quality and ensure the safety and well-being of workers. They can detect and measure hazardous gases generated in manufacturing processes, such as solvents, fumes, and toxic gases. As for process monitoring, ZnO gas sensors find application in various industrial processes where real-time monitoring of gas concentrations is required. This includes monitoring gas levels in chemical reactions, material off-gassing, and quality control in manufacturing processes. Lastly, they are also useful in leak detection. ZnO gas sensors are utilized in industries such as oil and gas, chemical, and refrigeration to detect and locate gas leaks. They provide early warning systems for detecting leaks of flammable, toxic, or harmful gases, helping to prevent accidents and ensure worker safety.

### 3.4. Indium Oxide (In_2_O_3_)

Indium oxide (In_2_O_3_) is an n-type semiconductor with a large bandgap (3.75 eV). In_2_O_3_ exists in two crystalline lattice structures: cubic and rhombohedral with the bandgap energy of 3 eV. Cubic indium oxide has a relatively high electrical conductivity, it is non-stoichiometric, and it is widely used in microelectronics [76]. The properties of In_2_O_3_-based gas sensors strongly depend on the preparation conditions, the electronic state of the indium, and the phase composition. In_2_O_3_ has found numerous applications in various fields, such as photoelectric devices, biosensors, solar cells, gas sensors, and high-transparency coatings [76].

Indium oxide (In_2_O_3_) gas sensors are used for gas detection, environmental monitoring, industrial applications, and in the automotive industry for emission control. Here are examples of environmental monitoring and industrial applications for In_2_O_3_ gas sensors. First, there is environmental monitoring and environmental remediation. In_2_O_3_ gas sensors play a role in environmental remediation efforts by detecting and monitoring hazardous gases in contaminated soil, water, or air. They assist in identifying pollution sources and assessing the effectiveness of remediation strategies. Then, there are industrial applications such as emissions control. In_2_O_3_ gas sensors are used in industrial sectors, including power plants, chemical plants, and manufacturing facilities, for emissions control. They monitor and regulate the levels of pollutants such as nitrogen oxides (NO_x_), sulfur oxides (SO_x_), and volatile organic compounds (VOCs) to ensure compliance with environmental regulations. As for industrial processes, In_2_O_3_ gas sensors help detect and measure gases generated during manufacturing, chemical reactions, and material off-gassing, ensuring process efficiency and safety. In addition, In_2_O_3_ gas sensors are employed in industries such as oil and gas, chemical, and refrigeration for gas leak detection. They provide early warning systems for detecting leaks of flammable, toxic, or harmful gases, helping to prevent accidents and ensure worker safety. In the automotive industry, they monitor and measure gases such as nitrogen oxides (NO_x_), carbon monoxide (CO), and hydrocarbons (HC) in vehicle exhaust systems, enabling compliance with emission standards and improving air quality.

### 3.5. Tungsten Oxide (WO_3_)

Tungsten oxide is an n-type semiconductor with a large bandgap (2.6–3.25 eV); it is one of the most used materials for MOXs gas sensors. WO_3_ has a very complex non-stoichiometric crystal structure and is available in various lattices (cubic, monoclinic, hexagonal, and orthombeic) [77]. The monoclinic WO_3_ (P2_1/n_) has been mostly studied for gas measurement applications. The thermochromic properties of WO_3_ make it useful for smart electrochromic displays and windows [78]. It also has found extensive applications in biosensors, gas, humidity, and UV sensors. The specific WO_3_ properties such as its reversible conductivity change, selectivity, biocompatibility, and high sensitivity make it suitable for biosensing applications [79].

The WO_3_ films may be produced in different phase (crystalline lattice), porosities, sizes, and thicknesses through various methods such as sputtering, PLD/CVD, wet chemical method, vacuum sublimation, electrochemical, and thermal oxidation [80]. The performance parameters of WO_3_-based gas sensors mainly depend on the preparation method and post-annealing operation [80]. Tungsten oxide (WO_3_) gas sensors are used for gas detection, environmental monitoring, industrial processes, and in the development of smart windows for energy-efficient buildings. Here are examples of environmental monitoring and industrial applications for WO_3_ gas sensors. First, there is environmental monitoring and air quality monitoring in particular. WO_3_ gas sensors are utilized in air quality monitoring systems to detect and measure pollutants such as nitrogen dioxide (NO_2_), carbon monoxide (CO), ozone (O_3_), volatile organic compounds (VOCs), and other harmful gases present in the ambient air. They contribute to assessing and improving overall air quality. Then, there are industrial applications and industrial processes. WO_3_ gas sensors are employed in various industrial processes for gas monitoring and control. They detect and measure gases generated during manufacturing, chemical reactions, combustion processes, and material off-gassing, contributing to process optimization, efficiency, and safety. WO_3_ gas sensors are also utilized in the development of smart windows for energy-efficient buildings. These sensors can detect changes in gas concentrations, such as carbon dioxide (CO_2_) levels, and trigger the windows to adjust their transparency or ventilation accordingly, optimizing energy usage and indoor comfort.

### 3.6. Copper Oxides (Cu_2_O and CuO)

CuO is a p-type semiconductor with a narrow bandgap of 1.2 eV. The copper oxide known as cupric oxide is black. Another copper oxide is the red-colored cuprous oxide (Cu_2_O), which is red in color and has a bandgap of 2 to 2.17 eV [81]. CuO has a monoclinic crystal structure.

CuO has been used for various applications such as batteries, biosensors, thin film transistors, sensors UV sensors, solar cells, humidity sensors, and gas sensors [82]. Cu_2_O-based sensors have been less addressed in gas-sensing applications compared to CuO [82]. P-type CuO semiconductors react differently compared to n-type metal oxide semiconductors such as SnO_2_, ZnO, WO_3_, and TiO_2_. The most important advantage of p-type metal oxides is their less temperature-dependent conduction at the high-temperature range compared to n-type metal oxides. The p-type metal oxides also tend to replace lattice oxygen simply with air. This property is useful in maintaining the stoichiometry of the metal oxide for the long life of the sensor. If used carefully, this advantage can maintain stability for a long time and increase the useful life of the CO gas sensor.

Thermal oxidation is a very popular method in the synthesis of CuO-based sensors due to its cost-effectiveness, simplicity, and good quality; it is, however, time-consuming. CuO has been used as a gas sensor to detect a variety of reducing and oxidizing gases [83]. Copper oxide (Cu_2_O and CuO) gas sensors are used for gas detection, industrial processes, environmental monitoring, and in renewable energy applications. Here are examples of industrial processes and environmental monitoring applications for copper oxide gas sensors. First, there are industrial processes and combustion processes. Copper oxide gas sensors are used in industrial combustion processes, such as in power plants or manufacturing facilities, to detect and monitor gases such as carbon monoxide (CO) and hydrocarbons. These sensors help ensure efficient and safe combustion. Copper oxide gas sensors also find application in the petrochemical industry for monitoring and controlling gas concentrations in various processes. They assist in detecting and measuring gases such as hydrogen sulfide (H_2_S), sulfur dioxide (SO_2_), and volatile organic compounds (VOCs) to maintain safe working environments. In addition, copper oxide gas sensors are also employed in renewable energy applications, such as monitoring gas emissions in solar cell manufacturing or detecting gas leaks in renewable energy storage systems. These sensors contribute to ensuring the safety and efficiency of renewable energy processes.

## 4. Main Sensing Properties of MOXs Gas Sensors

Sensitive response and recovery time, selectivity, the limit of detection (LoD), resolution, stability, and operating temperature are the major quality indices representing the performance of the gas sensor [84]. The sensitivity of the gas sensors is in a high degree dependent on the porosity of the sensing material, presence of dopants/modifiers, operation temperature, and crystallite size [85,86].

The LoD refers to the minimum concentration of the gas analyte that can be measured. It is a crucial parameter in determining the suitability of various gas sensors, including those that utilize MOS sensing materials, particularly for applications such as air quality monitoring. Table 1 illustrates the threshold limits of major air pollutants established by the European Union (EU) and the National Ambient Air Quality Standards (NAAQS) in the United States. These limits typically range from a few parts per billion (ppb) to a few parts per million (ppm). While studies cited in Table 2 indicate that MOS gas sensors can operate below these limits, it is important to note that these systems are currently in the research and development phase [63].

As the reactions often occur on the surface of the sensing material, controlling the size of the semiconducting MOXs material is one of the priorities to increase the sensitivity of the sensor. In this regard, nanocrystals have shown the highest sensing signals due to their high specific surface area and higher adsorption capacity [106,107]. H_2_ detection is now possible at the sub-ppm level due to the decrease in the size of the sensing material [108]. It has been also proven that TiO_2_-based sensors can detect humidity even at room temperature [109] at higher response and selectivity relative to H_2_S, CH_3_OH, and C_2_H_5_OH at lower temperatures [110]. Therefore, sensors have been developed with excellent mechanical stability, proper repeatability, fast response/recovery times with lightweight, and minimum energy consumption.

Selectivity defines the ability of the semiconducting layers to detect the target gases or a single gas in a mixture [84]. The selectivity of the MOXs gas sensors can be improved by surface modification or bulk doping with catalytic dopants to enhance the adsorption of target components [111,112]. Previous studies have shown that the sensing materials based on SnO_2_ and TiO_2_ can result in high-selectivity sensors, facilitating the detection of the target gas in a mixture.

Long recovery time is accompanied by the slow surface reaction rates which can be accelerated by MOXs doping with metallic catalysts such as Pd and Ag [113]. The response and recovery times depend on the working temperature. Fields et al. (2006) have developed a sensor based on a single SnO_2_ nanobelt for hydrogen detection and found that the response time declined from 220 to 60 s by elevating the working temperature of the sensor to 80 °C, while the recovery time showed a two-fold increase [114]. Landau et al. (2009) have explored the effect of temperature on the properties of the sensor and concluded that the response time and sensitivity of TiO_2_ nanofibers toward NO_2_ will be declined by temperature elevation, while the response time showed a decrement by increasing the concentration of NO_2_ [115]. In addition to the properties of the sensing material, the schematic of the sensors and their design and dimensions also play a decisive role in their performance [116].

In addition, the geometry and gap size of electrodes are the other important factors influencing MOXs-based sensors’ performance. Any change in the size of the electrode gap can alter the resistance of the device and hence the sensitivity of the sensor. Shaalan et al. (2011) investigated the impact of the SnO_2_ sensor gap size on NO_2_ detection and found that the selection of the electrode gap size depends on the concentration of the target gas. They also revealed that the electrodes with large gaps are highly sensitive to high NO_2_ concentrations [117]. On the one hand, the decrease in the electrode gap size can improve the selectivity of the sensing material for decreased gas concentration, and on the other hand, the selectivity can be improved by enhancing the width of the electrode line.

Previously, SnO_2_ and TiO_2_ have been synthesized with various morphologies. It was observed that the morphology can result in unique properties with a prominent role in their application. The following methods are often used for the synthesis of these compounds: electrochemical anodization [118], hydrothermal method [119], template-assisted synthesis [120,121], electrospinning [122], and matrix-assisted pulsed laser evaporation [123].

Long-term stability in the presence of siloxanes can decrement the accuracy and even makes it impossible to use MOXs-based sensors in platforms with a high siloxane load [124]. The lack of repeatability and stability is another drawback of the MOXs sensors [125]. The main problems of the low-cost gas sensors were summarized in [126]; they are related mainly to (i) issues related to the working principles of the sensors such as dynamic boundaries, nonlinear response, systematic errors, and signal drift as well as (ii) issues assignable to the external errors such as low selectivity and environmental dependence.

Multisensory approach, combining a series of interconnected sensors (sensor array) with machine learning algorithms, can be used to overcome these limitations. For gaseous phase analysis, such a system was named electronic nose, or e-nose, in similarity (even if rather relative) to the working principle of the mammalian olfaction system. Predictive models such as partial least square (PLS), support vector machine regression (SVM), artificial neural networks (ANNs), and others have been employed in the MOXs-based e-nose to identify and to estimate the intensity of the odor [46].

## 5. The MOXs Materials for Sensing Applications

The phenomenon of changes in the resistance of semiconductor sensor elements in the presence of an oxidative or reductive gas while operating at high temperatures is the basic principle of MOXs sensors operation [127]. These sensors convert one type of energy to another. That is, when an input signal (physical, chemical, or biological) is applied to a MOX sensor, it is converted to an electrical output signal. The MOX sensors have a simple design allowing their mass production and wild application; the most representative examples are overviewed in details in the next sections.

### 5.1. MOXs Sensors for Polluting Gases Evaluation

#### 5.1.1. NO_x_ Gas Sensors

Nitric oxide in the atmosphere is the main cause of acidic rains, leading to a high adverse impact on the environmental pollution in general and onto the respiration system of flora, fauna and humans in particular; hence, accurate outdoor NO_x_ content detection is a challenging analytical task. An atmospheric NO_x_ is a relatively inert mixture with a high combustion temperature. Solid-phase MOXs sensors are widely employed for the detection of NO and NO_2_ gases on the industrial and laboratory scales, but a limited number of commercial NO_x_ sensors can be found in the global market. In the laboratory scale, WO_3_, SnO_2_, ZnO, In_2_O_3_, and TiO_2_ are employed for NO_x_ measurements due to their low synthesis costs, proper reactivity with gas molecules, and good selectivity in a combination of materials [128]. A combination of oxides such as WO_3_ and ZnO with Ag, Au, and Pt can improve the sensitivity of the NO_x_ sensor. These oxides are naturally non-stoichiometric and have oxygen deficiency. The conductivity of these materials is examined based on the electrons provided by the additional metal. Upon exposure to gas at 200–300 °C, the amount of the electrons decreased due to the gas molecules of the intermediate reaction, which can increase the resistance of the sensor [129]. Industry and transportation are regarded as the two main sources of NO_x_. These gases cause nitrogen deposition. They can also result in cardiac and respiratory diseases in humans. Toxic gases such as NO_x_ from the vehicle’s exhaust and SO_x_ released from the industrial plants are in the class of primary pollutants. Secondary pollutants are not directly released, as they are produced during the reaction/interaction of the primary pollutants in the air [130]. This sensor has been used in types of research, such as the evaluation of diffusion mechanisms in different diesel engine conditions [131] and investigation of cross-sensitivities of NO_x_ sensors in SCR (Selective Catalytic Reduction) operation [132].

#### 5.1.2. CO_x_ Gas Sensors

The CO_x_ gas has no odor, color, or taste; that is, it is also called the “silent and invisible killer”. The presence of CO_x_ affects human health by decreasing the oxygen level of the blood, causing cardiac dysfunction.

Combustion, power generation, and the transportation sector are the main sources of CO. It can lead to headaches and cardiac failure in humans. Its long-term exposure can result in coma.

Fossil fuels, cement, and vehicles are the main source of carbon dioxide. This gas is the major cause of global warming and can affect the oxygen content of the blood in humans. CO_2_ detection has had increasing significance in the industry today, as its high concentration in the workplace causes respiratory diseases. On the other hand, it contributes to the greenhouse effect. Current CO_2_ sensors are based on hard ceramic materials that require high working temperatures (200–600 °C) and high energy consumption (200–300 mW) [133].

Moreover, an excess amount of carbon dioxide is harmful to our planet energy balance since CO_2_ absorbs short wavelengths reflected from Earth to space, thus elevating the Earth’s temperature, altering climate patterns, and causing floods, drought, and melting of ices in the poles [134]. According to the American Society of Heating, Refrigeration, and Air Conditioning Engineers (ASHRAE), the maximum CO_2_ concentrations for outdoor and indoor spaces should not exceed 350–800 and 1000 ppm, respectively [135].

Researchers today report different materials and classes of CO_2_ sensors such as solid electrolytes, semiconductors, mixed oxide capacitors, and carbonate-soluble polymers [136]. The solid-electrolyte CO_2_ gas sensor is highly useful due to its excellent sensitivity, low manufacturing cost, and proper selectivity. In recent years, NASICON (sodium (Na) Super Ionic CONductor) and LISICON (LIthium Super Ionic CONductor) are used mainly as active substances for CO_2_ measurement by combining the auxiliary phase of alkali metal carbonate [137]. The stability of the electrochemical sensor is its main problem as it changes over time, requiring recalibration [137].

#### 5.1.3. SO_x_ Gas Sensors

Combustion and power generation are two main sources of SO_x_ gas in atmosphere, which then becomes the main cause of acid rain that affects trees, rivers, and lakes. Exposure of the leaf to high levels of this gas (>100 ppm) destroys its tissue and also damages the edges and the area between the veins [138]. In addition, it causes health disorders in humans such as respiratory problems and impaired vision. Although the solid electrolyte-based sensors have been shown to measure effectively SO_x_ gas, the main problem is their insufficient stability [139]. On the contrary, the NASICON-based sensors being highly sensitive to SO_x_ remain also quite stable for a long time even in highly corrosive media [139].

#### 5.1.4. H_2_S Sensors

The H_2_S gas is colorless but malodorous, well water-soluble, corrosive and flammable in ambient conditions. It is poisonous already at sub-ppm levels, while the higher concentrations may cause a death of living beings. The main sources of this gas are geothermal activities (hot springs, natural gas, and side product of crude oil), landfills, organic decomposition of wastewater, and Sargasso algae [140]. According to the Occupational Safety and Health Administration and the Bureau of Labor Statistics, H_2_S is one of the most dangerous gases in the workplace [140]. International Public Safety Organizations have set exposure limits ranging from 1 to 100 ppm to limit olfactory disturbances caused by the natural and industrial production of H_2_S and to protect workers from acute and chronic exposure. Humans can smell H_2_S at low concentrations (0.5 to 300 ppb). At higher concentrations, this gas can cause serious injuries such as nausea, loss of olfactory ability, irritation of the nose and throat, and even death [141]. H_2_S is also present in environments where the humidity and concentrations of auxiliary pollutants (such as SO_2_ and CO in geothermal activity) are constantly changing. Therefore, it is necessary to have control and monitoring tools capable of operating in different concentrations and environmental conditions. Analytical methods such as gas chromatography and/or spectroscopy are commonly used to determine the concentration of H_2_S in the air [142]. Meanwhile, MOX sensors have high sensitivity, easy integration, and fast response in compact electronic devices, with high dependence on the relative humidity and limited selectivity. These devices mainly operate at high working temperatures (above 100 °C) [143]. Among the research studies in which this sensor is used, the following can be mentioned: quantitative and qualitative analysis of gas mixtures such as “hydrogen sulfide in air” and “methane in air” using temperature modulation of the metal oxide sensor [144], H_2_S sensing by all nanomaterials and different types of gas sensors [145], and the sensing behavior of CdS nanoparticles at room temperature [146].

#### 5.1.5. Volatile Organic Compound Sensors

Volatile organic compounds (VOCs) can also be detected with MOX gas sensors. Personal and home care products, building materials, as well as combustion processes (e.g., smoking, cooking, paint, cleaners, pressed wood items) constitute the most well-known indoor sources of VOCs [147]. In turn, traffic emissions, the burning of wood, oil and gas extraction, as well as emissions from industry constitute important outdoor sources. The detection of VOCs is also increasingly being used in health monitoring, medical diagnosis, as well as agricultural production and quality evaluation. Using the gas sensor array with MOX sensors [147], the representatives of volatile organic compounds—acetone, ethanol, and isopropyl alcohol (IPA)—as well as their binary and ternary mixtures in a simulated indoor ventilation system were examined. In the aforementioned publication, four metal oxide gas sensors created an electronic nose, which was subsequently employed in a flow-through system. Classifier and regression models built using backpropagation neural network (BPNN) were utilized for the qualitative and quantitative detection of VOCs. VOCs were mixed in various proportions; ethanol and isopropyl alcohol had comparable chemical and physical properties. In both cases, it was difficult to obtain quantitative outcomes. During network training, the Levenberg–Marquardt algorithm was chosen to estimate the quantity of VOCs in the mixtures. The BPNN-based model performed better when separating ethanol from IPA than the multivariate linear regression method. The accuracy of classification reached 82.6%.

Formaldehyde (HCHO) is an achromatic toxic VOC with a stimulant odor. Short-term exposure to HCHO causes headaches, while its prolonged exposure leads to asthma and pulmonary disorders [148]. In [149], Castro et al. reported a SnO_2_ nanowire-based formaldehyde sensor in which the addition of gold and platinum nanoparticles not only improved the sensor sensitivity but also has permitted the detection of HCHO at low working temperatures. In the same period, Descamps et al. proposed a method for measuring the concentration of gaseous formaldehyde by means of a nanoporous film doped with Fluoral-P [150].

The issue of the selective detection of hazardous indoor VOCs using metal oxide gas sensors relating to formaldehyde (as mentioned earlier) as well as benzene and naphthalene in ppb and sub-ppb concentrations with a varying background of ethanol was thoroughly discussed in [151]. MOX gas sensors with temperature-cycled operation and signal analysis based on pattern recognition can detect ppb-level VOCs with adequate sensitivity and selectivity, as shown in the aforementioned research. Even at varying concentrations of up to 2 ppm, hazardous target gases have been observed and identified in the ppb and sub-ppb range. The detection of naphthalene and benzene using MOX sensors was also described at concentrations in the ppb and even sub-ppb range both with and without interfering gases [152]. Results exhibit the high responsiveness (relative change in conductance) of a commercial thick film sensor, reaching approximately 15% for 500 ppt benzene and 137% for 40 ppb naphthalene, also when ethanol was present as an interfering gas (2 ppm). The absolute change of sensor conductance in the case of naphthalene is constant both in the presence and absence of an interfering gas.

Other VOCs were also detected using metal oxide sensors. A paper by Wang et al. [153] showed the possibility of detecting, xylene, trimethylamine, triethylamine and (as mentioned earlier) formaldehyde using metal oxide sensors with various dopants, e.g., Ni, Pt, Au, Fe, Ce, and Cr and with different surface morphologies, e.g., nanolamella, nanobelts, nanorods, nanoarrays, nano-pompon, nanosheets, microsheets, and micrograss.

## 6. Application of MOXs e-Noses for Environmental Monitoring

The application of electronic noses in the environment includes the following main categories: (i) air quality parameters analysis; (ii) water quality monitoring; (iii) process control; and (iv) verification of odor control systems efficiency [154].

In each of the mentioned applications, an e-nose can be used as an alternative or auxiliary method for traditional analytical methods. Concerning air quality monitoring, an MOXs-based electronic nose can be an alternative to gas chromatography in assessing the presence and concentration of various pollutants [155]. Therefore, an e-nose can be employed for process control instead of costly analysis approaches which are often time-consuming as well. For instance, unwanted anaerobic processes in a compost plant can be achieved by combining temperature, oxygen level, pH, and microbiological measurements [156]. As an alternative, similar data can be obtained by a trained e-nose (specifically for the composting process) at lower costs and complexity in a completely descriptive manner. In addition to serving as a complementary or alternative method for the analytical approach, e-noses are the only tools capable of evaluating and classifying odors. That is why proper sensing methods are utilized to quantify the odor.

### 6.1. Evaluation of the Air Pollution

Pollution monitoring is a key step in the management of air quality. The concentration of the pollutants is measured in the reference stations using accurate analytical tools comprising energy-demanding and heavy equipment. Therefore, the number of these stations is limited due to their high operation and maintenance costs. These stations are preferably located in urban areas, although they may be far from the main source of pollution. In rural, less-populated, and remote areas, there are no pollution measurement stations; hence, no pollution data are available [7]. It has been estimated that the use of low-cost and energy-efficient sensors complying with the quality standard of AQD can decrement the minimum number of stations by 50% [157]. Currently, the best alternative to these costly devices for monitoring air pollution is the e-nose comprising an array of sensors and portable devices to measure the pollution in developing cities [158,159,160]. The first step in the development of e-noses for environmental applications is reducing the cost of sensors. In addition to low cost, these sensors must be independent, feasible, and precise. Their size, weight, and energy consumption rate should also be small [161]. In this regard, MOXs sensors are the most appropriate choice.

Most of the studies have emphasized air pollution as the largest environmental hazard throughout the globe, which is almost intangible for most people. Based on the latest data from the World Health Organization (WHO), air pollution leads to 7 million death cases each year [162]. Despite the huge extent of the low air quality, it cannot be observed with the naked eye, and people are unaware of the air quality they are exposed to. According to the WHO, air pollution can intensify asthma, especially among children, and it is also the major cause of mortality in cardiovascular disease patients including stroke, cancer, and chronic respiratory disease [163]. According to the above-mentioned discussion, air pollution is currently the largest health hazard. Some of the main atmospheric pollutants and their influence on the environment and human health as well as detection with MOXs sensor arrays will be discussed below, among them: carbon mono- and dioxides (CO and CO_2_); sulfur dioxide (SO_2_); a nitrogen oxides group (NO_x_); and several volatile organic compounds (VOCs).

Recently, the application of employed an e-nose equipped with MOX sensors connected to a smartphone (by Bluetooth) to measure the air quality was reported by Arroyo et al. [162]. The detection ability of the device for various concentrations of NO_x_ compounds (one of the main air pollutants) using a perceptron ANN in an application specifically developed for smartphones has been estimated. Several tests were conducted under various relative humidities to assess the effect of humidity on the detection performance of the gas sensors, and it was demonstrated that high humidity can dramatically affect the device performance, especially in the detection of low levels of pollutants. The authors have planned that future works should be focused on the improvement of the reliability of MOX sensors in the presence of humidity to achieve proper prediction for various air pollutant contents.

**Figure 7:**
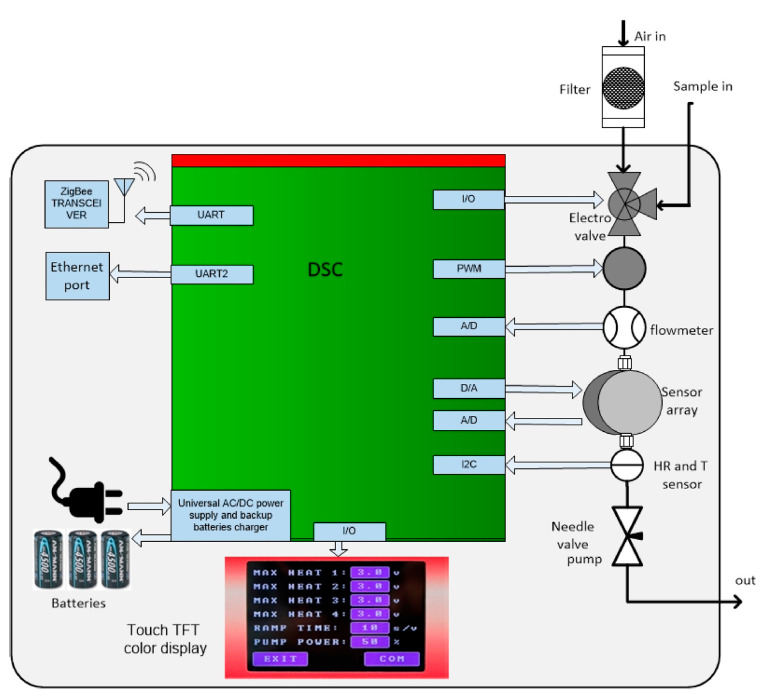
The schematic representation of the WiNOSE device. Reprinted from Ref. [7].

In [7], Sayago et al. applied two prototypes of e-nose for environmental applications based on low-cost sensors. The sensors were based on nanostructured (nanofiber) tin oxide and implemented in a WiNOSE 50 e-nose and commercial sensors installed in WiNOSE 6.0. These sensors were applied to assess low levels of NO_2_ in a controlled atmosphere at room temperature. The general design of these two devices was very similar, as shown in Figure 7. The first one had laboratory applications, while the second one can be manually used in the ambient environment. In the WiNOSE 5.0, three tin oxide nanosensors were used, while WiNOSE 6.0 encompassed eight advanced commercial MOX microsensors.

The sensors showed low error, especially in the temperature range of 50–100 °C, and there was even lower error at room temperature. Moreover, a combination of both sensors in a multi-linear calibration led to better results. The PLS method exhibited very good performance at 50 °C, but its error rose with the temperature elevation. Nanofiber-based sensors showed better performance at lower temperatures as compared with MOX sensors. MOX sensors, alone, showed no considerable response at temperatures below 250 °C. Regarding the obtained results, it seems that the MOX sensors, alone, are not suitable for NO_2_ measurement due to their high error, and they should be used in combination with other sensors. The MOX sensors, however, exhibited proper performance at higher temperatures.

In research presented by Vuka and coworkers [164], the six metal oxide sensors and an ultrasonic anemometer wind sensor were employed to find the origin of hexane gas. The sensors were integrated in a mobile robot in a Gaussian regression domain approach with an active search algorithm (gas source localization, GSL) learned to find the gas-polluting gas source. In fact, over the last two decades, various studies and methods have been conducted to find the gas source in which robots use the two principles of anemotaxis and chemotaxis and follow the gas in the opposite direction [165,166]. In some cases, the gas source location is modeled by the probability distribution using complex algorithms [167]. Finally, the modified version was reported by Schmuker et al. in [168]. In [155], however, the robot seeks the environment and builds a possible model for the distance to the source. The environment was considered known by authors, and it was assumed that there is only one source of gas in the environment. The robot moves and records gas concentration and calculates the wind data for a certain period to estimate the distance to the source. The routing strategy involves guiding the robot by the amplitude of the concentration signal to the direction where the gas concentration is expected to reach its maximum. This technique was performed with a success rate of 67%. In this strategy, routing should be improved to enhance the success rate of GSL. Regarding the efficiency of MOX sensors, it is possible to use the robot in environments with multiple gas sources to identify several gas sources.

Previously, Helli et al. have employed MOXs sensors to detect the presence and concentration of NO_2_ and H_2_S in CO_2_-containing atmospheres with certain levels of humidity [169]. It was shown that the e-nose can correctly detect the composition of the test mixture. However, it was stated that this detection is under the influence of CO_2_ and humidity and is only accurate when the mentioned parameters are known.

In [170], Negri and Reich have utilized an electronic nose with MOXs sensors to detect CO, methane, isobutane, and ethanol in atmospheres containing disturbing gases. The developed system managed to detect correctly 85% of the concentration of various compounds with an error below 10%. These results were highly satisfactory, even if the concentration of the analyte was very high compared to the levels commonly encountered in ambient air (500–1000 ppm).

Wolfrum et al. have used fourteen MOXs sensors for the identification and quantification of several VOCs, such as acetone, toluene, and isopropanol even at very low concentrations (ppb) [171]. To establish a linear correlation between e-nose responses and the concentration of VOCs, the data were pre-processed and then used for quantification. A linear correlation was obtained between the actual and the estimated concentrations.

The ability of an MOXs-based e-nose to classify malodorous air was reported by Sironi et al. [172]. The receptors of the e-nose were constantly exposed to the odor of a compost plant. Two e-noses, each with six MOXs sensors, were tested in parallel and trained with samples collected at a supervised compost plant. The e-noses analyzed the air every 12 min for 4 days. The sensor response process was then explored using an ANN algorithm to classify the analyzed air into different olfactory classes intended for training. The obtained results of the e-noses were compared with recordings of the residents. The measurement accuracy index of the e-nose in detecting the presence of odor was calculated as a percentage ratio between the number of correct classifications and the total number of measurements, which was equal to 72%.

Burgués et al. have used a pure analyte signal to estimate the limit of detection in temperature-modulated MOXs sensors [173]. The limit of detection (LOD) is defined as the minimum analyte concentration that can be measured reliably. The LOD is an essential indicator for the reliable detection of harmful levels of toxic pollutants (such as SO_2_, NO_2_, and CO) and may be influenced by environmental interactions such as humidity, which are commonly present in the detection of chemicals. The application of these cases on temperature-modulated MOXs sensors is a challenge due to the nonlinear multivariate sensor response, cross-sensitivity to humidity and temperature, dissimilar quantitative conditions, and sensor drift. However, some researchers have estimated LOD in MOXs sensors using alternative approaches [174]. In [164], ten MOX sensors (FIS SB-500-12 and FIGARO TGS 3870-A04, five of each type) were used to detect CO in varying air humidity (20–80% RH) at a constant airflow of 1 l/min. It was shown that at low concentrations (less than 50 ppm), the MOX signals could be fitted with linear models and that the homoscedasticity condition was met; moreover, cross-sensitivity to moisture was the major factor in reducing the LOD of MOX sensors. According to the experimental treatments, it is recommended to conduct this experiment in multiple air streams to achieve more comprehensive and reliable findings.

In the research of the Lilienthal group, a cluster analysis approach based on the use of density peaks was employed for outdoor gas detection by means of e-noses [175]. The model proposed by Fan et al. (2017) [175] which can identify the number of different chemical compounds was employed. The samples collected from indoor and outdoor environments by a mobile robot equipped with a set of commercial MOXs sensors were analyzed. The results showed that the exact classification can be achieved with low sensitivity to the choice of the only free parameter (i.e., proximity size), which is used to estimate the density in the clustering process.

Previously, various solutions have been proposed for the problem of gas detection in uncontrolled environments. For example, Vergara et al. developed a gas detection system using SVM [176]. It was found that the classification result of this method is influenced by the strength of the wind flow and the distance between the sampling site and the gas source. Several chemical compounds as target analytes under a series of different environmental conditions were tested in a wind tunnel, in which the e-noses consisting of six commercial Figaro MOX sensors were located at different distances from the gas outlet. The authors stated that a robust gas detection system must be trained in a comprehensive environment, which is not always possible in practice. As a development work, Fonollosa et al. exposed an array of eight Figaro MOX sensors to a mixture of dynamic gases [177]. In this study, gas plumes were naturally mixed along with the turbulent flow in the wind tunnel. The inhibitory SVM was implemented as a tool to classify chemical components in dynamic turbulent mixtures. Based on the results, it was concluded that the high concentration is not enough to achieve optimal classification, and the classifier training would consider data at low concentrations as well.

With the prospect of biological olfactory imitation, e-nose technology has its advantages and limitations. Currently, most of the reported sensors can measure and detect gas at different ppm levels. In some cases (e.g., breath detection and explosive detection), however, high-sensitivity sensors are required. Therefore, finding an effective way to increase the sensitivity of the sensor is vital. Among the different solutions in regard to the enlargement of the surface of the sensing materials [178], the pressure-regulated piezoelectric effect application [179], the strain-induced additional piezoelectric gate voltage implementation [180], etc. have been suggested previously.

High sensitivity, proper stability, working at room temperature, fast recovery and response are among the vital factors in the e-nose technology. The point is that strong adsorption or chemical reaction may result in proper selectivity and/or sensitivity, but it will hinder the sensor recovery [181]. The sensor baseline may alter due to environmental conditions such as temperature, humidity, and pressure, which may pose further difficulties in the online evaluation. Some additional temperature, humidity, and pressure sensors may be added to the e-nose for calibration. Moreover, the metal–organic frameworks (MOFs) coatings or molecular layers on the sensing material can be effective in reducing the influence of the interfering gases [182]. Sampling the real analyte is a difficult task, and it must not lead to a deviation in the results. In this regard, adsorbents can selectively collect analyte and pre-concentrate it to further facilitate the detection by e-nose. MOFs or covalent organic frameworks (COFs) have tunable pore sizes which can selectively adsorb a specific type of gas [183].

While dealing with both indoor and outdoor gas analyses, the synchronous measurement of the signal of an array of sensors at high precision is still a challenge that can be resolved by the following solutions: (i) a combination of a switcher and source-seeking method, (ii) using a timer for the simultaneous operation of several synchronous source-seeking methods, and (iii) a specifically designed measurement system. The first approach is not expensive and involves the use of a switcher, which delays shifting from one sensor to another for precise measurement of the data. The second method is synchronous, but its precision is lower than the source-seeking method in addition to being much more expensive than the first method. The third method is the most suitable approach with the least costs [182]. The fluid of the analyte gas also affects the sensor recovery. The performance of the e-nose can be significantly improved by proper design of the structure of the e-nose housing [184].

Regarding the above-mentioned discussion, the system should be first enhanced and calibrated depending on the type and location of the device; then, the e-nose can be utilized for the intended goal. Regarding the advantages and methods to resolve the limitations of the e-nose system, it can be employed for precise detection of the pollutants and the source of pollution in remote regions for a long time.

### 6.2. Pollution Evaluation in Water and Wastewater

The drinking water is easily accessible to all people in developed countries. The drinking water is, however, exposed to permanent threats due to the uncontrollable release of industrial wastes, which can result in microbial storms. In this context, frequent evaluation of the quality of water and the precise management of water resources are essential factors to ensure the supply of clean and safe water for humans [185] and the determination of the microbiological and chemical contamination [186]. The daily activity of humans has led to the discharge of a huge amount of pollutants into the environment including the seas and oceans which can seriously threaten marine life, causing continuous destruction of the oceans and seas as well as the shores [187].

In many cases, online precise tools are required for the analysis of the wastewater and water, which can be realized by the use of chemical gas sensors as they can present information about the condition in a short time. They can be used in the quality control of food products, environmental monitoring, and diagnosis of human and animal diseases. Their adaptability can be also assigned to the various materials used in their structure.

Wastewater systems, wastewater sludge processing plants, pumping stations, separation, and waste treatment and recovery units play a decisive role in environmental protection. They, however, pose a potential risk, the failure consequences and smell inconvenience can be reduced or eliminated by ensuring the correct performance of the system and taking preventive measures (for instance, when observing a change in the input quality) [103]. Wastewater treatment plants reduce the wastewater contamination before its release into the receiver unit. The processes occurring in the wastewater treatment plant are directly related to the release of bad smells [100]. Urban wastewater generally includes consumed water in the homes and public organizations, industrial wastewater, rain, and leaked water (from the pipes).

Wastewater treatment plants can be mentioned among some of the most troublesome sources of volatile air pollutant emissions. Any wastewater treatment process, especially those involving anaerobic conditions, contributes to odorant emissions. A survey of technical personnel at 100 wastewater treatment plants in Germany indicates that the predominant sites of odorant generation are mainly wastewater pretreatment processes (sand traps, settling tanks) and the processes associated with sludge treatment (sludge thickening and dewatering) [188].

The various processes contribute to significant levels of odor concentration. According to Kosmider et al. [189], the odor concentration level at the influent of wastewater and mechanical treatment facilities is 30–1000 ou_E_/m^3^; biological treatment is associated with concentrations of 5–120 ou_E_/m^3^, while sludge treatment is 100–1,000,000 ou_E_/m^3^.

Wastewater is a source of formation of a wide range of volatile organic compounds, so it is difficult to perform a comprehensive study of their chemical composition, and it is even more difficult to determine their synergistic effect on odor concentrations [190]. They usually include sulfur or nitrogen compounds, organic acids, aldehydes and ketones [191].

Concentration levels of individual pollutant components, such as hydrogen sulfide, can provide an indicator of odor nuisance in the air [192]. Gostelow et al. [188], in a study conducted for 17 wastewater treatment plants, determined the dependence of hydrogen sulfide concentration on odor in the range of 102–107 ou_E_/m^3^. This allowed them to derive the equation c_od_ = 38,902·c_H2S_^0.6371^, describing the aforementioned relationship with a coefficient of determination of R^2^ = 0.69. In contrast, in a study conducted at the University of Hertfordshire, UK, at 10 wastewater treatment plants, no statistically significant relationship was found between the concentration of hydrogen sulfide in wastewater and TON (Threshold Odor Number) in the ranges 0–4 × 10^3^ and 0–4 × 10^5^ ou_E_/m^3^ [96].

The possibility of MOXs-based sensory systems for pollution evaluation in water and wastewater analysis has been demonstrated previously in several research studies (Table 2). The most common of these were studies on the possibility of using the array to identify odors due to the location of their formation in a wastewater treatment plant [92,94,95]. In several cases, it was possible to correlate the response of the sensor matrix with parameters such as BOD, COD, total suspended solids (TSS) and turbidity [92,93]. Other studies indicate the possibility of detecting episodes indicating the presence of unnatural contaminants that can interfere with the biological treatment process [97,98,99]. MOX sensors are less susceptible to the temperature and moisture content of air samples above the wastewater surface than, e.g., polymer sensors, so it is more advantageous to use them in this type of solution [94]. MOX matrices were also used to evaluate odor concentrations near the boundary of the plot on which the treatment plant is located and near the nearest residential buildings [95].

Onkal-Engin et al. [92] evaluated the samples collected from a wastewater treatment plant by an array of gas sensors and classified the results using an ANN. Networks with 12 input neurons and two hidden layers of 12 neurons each were used for analysis. For the study, air samples were taken from several stages of the process: pretreatment, sludge tanks, biological treatment and wastewater outlet of the treatment plant. A total of 144 samples were collected for classification. Based on authors findings, this classification method can be utilized for the general classification of the samples, and the e-nose can monitor the BOD. As a result, a high correlation for each type of odor was obtained, reaching R = 0.99 [92].

In another study, three measurement stations with EOS25, EOS28 and EOS35 analyzers were deployed near a wastewater treatment plant, while each station consisted of six MOX sensors [95]. The sensor chamber was maintained at 50 °C, through which an air flow of 150 cm^3^ min^−1^ was passed. PCA (principal component analysis) was used to analyze the data. The purpose of conducting the measurement was first to identify the source (process) of malodorous substances and then to determine their odor concentration. As a result of the study, treatment plant processes were divided into three classes, which were related to wastewater, sludge and neutral ambient air. For odor concentrations of 100–150 ou_E_/m^3^, the accuracy of the classification was 0.95. The correlation between odor concentration and matrix response in the range of 20–80 ou_E_/m^3^ was also determined with an accuracy greater than 0.9.

Attempts were also made to determine the relationship of volatile air pollutants with wastewater parameters such as COD, suspended solids, turbidity. However, the correlation coefficient proved to be statistically insignificant (R = 0.52–0.67) [93]. On the other hand, another study conducted by Onkal-Edgin et al. [92] indicated that it is possible to correlate BOD5 with readings from a sensor array system. High correlation (R = 0.91) was obtained for the test sample, while the RMSE totaled 0.07. This represents a very good result despite the variable composition of the wastewater. The advantage is that the result is obtained almost at the moment of measurement, while standard BOD5 analyses take 5 days.

Systems with a sensor array are a very good tool for continuous control of the wastewater treatment process. Such a station that detects and alerts to the presence of abnormal contaminants has been installed at Cranfield University’s municipal wastewater treatment plant [97,98,99]. The measurement system consists of an initial mixing chamber connected to a flow chamber, where samples of wastewater headspace were taken for analysis over a period of six months with a five-minute interval. In addition, measurements of total organic carbon (TOC) were taken. Contaminants (such as oil) were added to the wastewater to simulate the presence of industrial pollutants. During the measurement period, there were also unidentified pollutants resulting from accidental discharges into the wastewater. The system accurately detected the presence of additional contaminants not naturally present in the wastewater composition. Basing on the idea of adding contaminants into the sample, experiments were conducted with different soils polluted by hydrocarbons and analyzed by an MOX sensors matrix [193,194]. Applied data analysis using ANN enabled recognizing soil polluted by petrol and diesel in samples of different soil type and origin.

A system for the classification of stages of wastewater treatment processes and full-scale WWTP (particular devices) was designed and presented by Łagód and coworkers [103,104]. The mentioned system was based on a gas sensors matrix consisting of 17 sensors by Figaro. For dimensionality reduction and preliminary data visualization, principal component analysis was applied [103] as well as the t-SNE method [104]. Basing on a distance matrix in multidimensional space, the k-means method (non-hierarchical cluster analysis) was used to find homogeneous clusters of data [103]. For evaluating the discretization potential of the collected multidimensional data enabling to seek general relations and the relationships between groups of data in multidimensional space, the k-median method was used [104]. As a simple predictive model with sufficient accuracy of about 98%, a decision tree model was used [103], while a more advanced random forest model allowed achieving an accuracy of 100% [104] basing on an analysis of 185 multidimensional datasets and readouts from 17 gas sensors.

A full-scale WWTP featuring an electronic nose system to characterize water quality parameters and odor concentration of wastewater emitted from different treatment devices was also developed and tested [105]. The applied sensors matrix consists of 32 gas sensors. Together with an analysis of headspace by the mentioned MOX matrix, an analysis of odor and water quality indicators (COD, AN, TN, TP) was performed. Alongside diverse feature extraction methods, support vector machine and linear discriminant analysis were applied as classifiers to distinguish samples, which stated the best recognition rate of 98.83%. Partial least squares regression was applied to complete the next step of data analysis, giving R^2^ at a level of 0.992. As the final step, ridge regression was used to predict water quality indicators and odor concentration with the RMSE being less than 0.9476 [105].

According to a study by Nake et al. [94], for measuring volatile air pollutants emitted by wastewater treatment plants, MOX sensors perform better than conductive polymer (CP) sensors. Their study at a wastewater treatment plant used a commercial Pen2 meter (WMA Airsense Analysentechnik, 10 sensors) and a Cyranose 320 (CyranoseSciences 32 CP). Measurements were taken for 8 days at five treatment plant locations. Statistical analysis of the obtained PCA results performed for the CP sensors showed high variability in the results, which was most likely due to their sensitivity to humidity (coefficient of determination to relative humidity R^2^ = 0.83–0.95) and air temperature (R^2^ = 0.86–0.91). In contrast, the results from the MOX sensors were more stable, as evidenced by a lower coefficient of determination to relative humidity (R^2^ = 0.64–0.89) and air temperature (R^2^ = 0.76–0.86).

Basing on readouts from a matrix of eight sensors by Figaro analyzing the headspace air from a laboratory sequence bath reactor with activated sludge, Guz at al. [48,100] present a possibility of obtaining a high correlation between measurements of odor nuisances by e-nose and the reference method: dynamic olfactometry. During the experiment, an Ecoma TO-7 olfactometer was used to measure the odor concentration, according to EN-13725:2007. For processing the multidimensional data from the gas sensors matrix, the authors trained an artificial neural network based on multilayer perceptron. The learning quality of the mentioned MLP ANN was 0.97 when testing and validation were 0.88 and 0.98, respectively. The coherence of the odor concentration assessed by dynamic olfactometry and predicted using a e-nose consisting of MOX sensors and the ANN method was at a high level (R = 0.85) [100]. This same matrix of MOX sensors by Figaro was used for the classification of malfunction in processes of wastewater treatment conducted in a laboratory sequencing batch reactor with activated sludge [102]. During the experiment, raw wastewater and treated wastewater after stable and efficient purification processes were distinguished. An important part of the investigation was to check the possibility of detecting the appearance and deepening of purification processes malfunctions, and after removing the breakdown, the restoration of proper purification conditions. The detection accuracy of all of the mentioned individual states was 78.04% [102]. Applying this same matrix consisting of eight MOX sensors by Figaro and analyzing the long-term experiment conducted with SBR and activated sludge, a correlation between the standard wastewater pollution indicator and multidimensional signal from a sensors matrix was established [101]. A high correlation (r) was achieved for the following pollution indicators: COD (0.988), TSS (0.938), turbidity (0.940), N-NH_3_ (0.978), N-NO_2_ (0.0.869), N-NO_3_ (0.958), and VOC (0.98), while the worst results were reached for pH (0.554) [101].

In [186], the use of metal oxide gas sensors embedded in an array for analysis of the complex volatile fingerprint of the water and wastewater samples was investigated. The gas chromatography technique along with solid-phase microextraction was employed as a reference method for a better understanding of the responses of the sensors and their relationships with a specific class of VOCs. The data from the employed GC-MS and a small sensor system (S3) were treated by principal component analysis (PCA) and artificial neural network (ANN) methods with respective classification accuracies of 95.84 and 97.62%.

An interesting approach to detect pollutants in the water samples using a wireless manual e-nose was adopted by Lozano et al. [195]. Two years later, the same authors reported a portable e-nose prototype equipped with an IEEE 802.11 transmitter–receiver for wireless connection ready for distributed measurements in an extensive network of the e-noses [196]. The developed system included pumps, electronic valves, and chargeable batteries capable of working with several resistive microsensors. The prototype was presented by measuring cycles of 60 min of adsorption and 540 s of desorption. It was shown that the developed system can reliably detect and differentiate water pollutants. Using PCA and ANN, the correct differentiation and detection were achieved in more than 90% of the samples with various component concentrations at the level of 1 mL in a 20 mL vial at 16 °C.

In [197], Tonacci et al. have implemented an e-nose system in an autonomous underwater vehicle (AUV) to control oil leakage in a protected marine region (Tuscany Islands and Cetacean). The device encompassed a sensing section (including photoionization detectors), an inlet, an air treatment unit (including an electronic pump and a valve), and smart electronic devices (based on Arduino mega 2560 board). A computer was also considered to collect the data. ANN was utilized to detect the pollution level (regardless of its source) and detect the substances in a series of known compounds. Despite the proper accuracy of the classifiers, the responses were unreliable when the system was exposed to relative humidity above 70%, suggesting the considerable limitation of the mentioned tool. The same research group presented two other extensions of this application [198,199].

In a work of Baby et al., the e-nose was employed to investigate the possibility of pollutants and pesticides detection in surface water [200]. An e-nose equipped with two MOXs and QCM (Quartz Crystal Microbalance) sensors was used, and this study proved that the e-nose could detect pesticides and differentiate among them.

Another application of e-nose technology for water quality monitoring is the detection of microorganisms. In [201], Bastos and Magan detected microorganisms responsible for malodorous substances in the waters. To prevent bad smell and hence the undesirability of water by microorganisms, the presence of these microorganisms should be detected in a short time. To this end, an e-nose was utilized to analyze the headspace of the microorganism-inoculated water. The possibility of detecting the presence of Streptomyces after 24 h of their growth was demonstrated. This tool also managed to differentiate non-contaminated water from the Streptomyces-contaminated water at the concentration of 102 spores/mL using PCA. The Lozano group has applied an e-nose with MOXs sensors to analyze the contaminated water samples in the laboratory as well as the samples collected from a wastewater treatment plant [202]. This e-nose was employed for the measurements of the headspaces over the samples. The data were then analyzed by PCA, and good discrimination was obtained for the contaminated laboratory waters. The system also could classify the analyzed samples using Radial Basis Function Networks (RBFs), achieving 100% correct classification for the laboratory samples. Moreover, this system managed to correctly classify 90% of the wastewater plant samples, thus demonstrating the suitability of the MOXs-based e-nose to monitor the quality of the water in wastewater treatment plants.

In another research study reported by Sohn et al., the quality of the contaminated waters was assessed. The study aimed to use an e-nose for the assessment of the volatile solid substances’ concentration in contaminated waters through the sample headspace analysis [203]. However, the obtained results have indicated that despite the linear correlation between the volatile solid substance content with other physical properties of the liquid, this correlation was not valid for the smell concentration estimation. Nonetheless, the e-nose managed to estimate the smell concentration of the air in contact with the contaminated water after proper training using the ANN technique, and the correlation coefficient of 0.98 was obtained between the smell concentration of the estimated and real smell concentrations.

Thanks to recent technological progress, researchers have found novel solutions in the field of potable water quality monitoring. For instance, the emergence of the Internet of Things (IoT) has been shown to be a prominent tool in a research of Climent et al., who employed IoT and developed a portable low-cost e-nose named as the multisensory olfactory system (MOOSU4) for bottled water quality control. The possibility of further extending the developed system for seawater assessment was also shown [204]. The volatile compounds, such as dimethyl sulfide, dimethyl diselenide, and sulfur were detected at the accuracy of 86%. Based on these results, the developed system can be applied for the monitoring and control of a wide range of VOCs.

As hydrocarbons are the major source of marine contaminations, some new approaches have been developed in this field. For example, in [205], the AlphaMOS e-nose combined with chemometrics to detect 444 samples comprising 40 μL of petroleum-derived products (PDPs) such as gasoline, paraffin, and diesel fuel have been utilized. This system managed to correctly detect the presence/absence of PDP in all cases and various PDPs in 97.9% of the cases.

The marine and shore ecosystems are under the continuous threats of human activities (ships, industrial discharge, etc.) which can result in serious and irreversible consequences for these regions and their wildlife. Various innovative funding bodies and solutions, including national and international projects, research teams, and scientific articles have been presented in the past decades [206]. These solutions are useful in the marine regions (especially the protected zones) in such a way that even detection of the low levels of the pollutants can be responded to by fast and effective measures, preventing environmental disasters. In this regard, the application of the low-cost, fast, reliable, and sensitive tools is of crucial significance; therefore, the use of e-noses seems to be necessary. In recent years, several products have been developed based on this technology to monitor seawater. The conductive polymer is among the first and most applied technologies [97,207] with promising results. However, such sensors are highly dependent on the environmental conditions which can affect the detection of the device. Experimental adjustment is also important, as the sensors should be properly cleaned after each measurement [205]. Currently, the main limitation is the need for a populated database to train the related detection algorithms. This drawback has been successfully resolved by the recent technological advancements. Photoionization detectors are one of the other methods in this field with fast responses [198]. They, however, suffer from significant problems in high-humidity media. The other solution was also proposed which requires tight control of the environment for a steady signal [196]. Hybrid approaches including a biological section comprising human olfactory receptors, as well as a technological part, were successfully employed by Son et al. in [208]. An e-nose can detect specific compounds. The development of such devices for the new technologies such as IoT, as mentioned by Climent et al. in [101], can open new horizons in the environmental fields, in particular, the monitoring and control of the seawater.

### 6.3. Commercial MOX e-Noses in Environmental Engineering Applications

There are numerous e-noses available on the market today, with a wide range of applications in different markets and industries, including environmental engineering. Moreover, they represent a wide range of sensor types, some of which also include MOX sensors. As e-noses are used increasingly often and the demand for them is growing, the companies producing commercial e-noses are constantly developing new prototypes that use other technologies and sensors but also have modules for increasingly advanced statistical data analysis. The above-mentioned facts also contribute to the use of commercial e-noses as part of scientific research.

The paper [209] investigated the correlation between microbial activity and odor emissions from municipal solid waste during the anaerobic digestion process at a full-scale wastewater treatment plant. For this purpose, measurements conducted by gas chromatography coupled to mass spectrometry and analysis of air samples using a PEN2 e-nose (Airsense Analytics) equipped with 10 MOX sensors were used. By means of the data obtained from the seven selected sensors, an $OD_{20}$ regression (oxygen measurement rate) vs. measurements from the e-nose using the PLS method was obtained, for which the coefficient of determination was $R^{2}=0.99$, while the cross-validation regression coefficient amounted to $R_{cv}^{2}=0.98$. In turn, lower coefficients were obtained for the PLS regression for the chromatograph data, namely $R^{2}=0.95$ and $R_{cv}^{2}=0.78$.

The PEN3 e-nose (Airsense Analytics) also consists of 10 MOX sensors, according to the manufacturer’s recommendations, and it can be used for odor testing in wastewater treatment plants and composting plants, filter inspection, leaks, exhaust emissions, and fire warning, among other applications. In the work [210], the PEN3 e-nose was used in conjunction with a Cyranose 320 (32 composite sensors) to identify the source of odors in solid waste composting plants. The data were classified using linear discriminant analysis (LDA), and for those obtained with PEN3, 86.7% correct classifications were obtained, while for the Cyranose data, it was only 53.3%. The best percentage of correct classifications was obtained by means of several selected sensors (four MOS and two CP) from the e-noses used.

The paper [211] used a Fox 3000 electronic nose (Alpha M.O.S.) comprising 12 MOX sensors to measure odor nuisance. Its performance was compared with an Aromascan A32S electronic nose (32 conducting polymers sensors) and an experimental one built with commercially available sensors (6 MOX sensors). For both commercial e-noses, a very high correlation coefficient (r=0.99) was obtained between the measured odor intensity and the odor intensity calculated using ANN. These are similar results, despite the fact that the Aromascan e-nose had more than 2.5 times the number of sensors.

The KAMINA electronic nose with an MOX sensor microarray was used in the work [212] to recognize samples of clean water and water contaminated with ammonia or chloroform. For this purpose, an LDA model was built on two measurements of flowing water; the model managed to recognize clean water samples (100% correct classifications), but ammonia-contaminated water was not recognized for any of the observations, while chloroform-contaminated water was recognized in 3% of cases. The authors attempted to improve the quality of the model by including data from 12 measurements taken in the headspace experiment. In the case of this model, the classification quality of clean water remained unchanged, while ammonia-contaminated water was recognized in 54% of observations and chloroform-contaminated water was recognized in 41%. Thus, it was concluded that it is possible to use data taken under different experimental conditions to improve the quality of classification.

Schreiber also used commercial KAMINA and MOSES II e-noses in his doctoral thesis [213]. They were used to investigate the perceived indoor air quality and to detect the presence of fungi in indoor air. MOSES II is an e-nose containing a set of sensors with quartz microbalance (QMB), SnO_2_ MOX sensors and amperometric gas sensors (AGSs). Measurements from the two electronic noses enabled to determine the presence of mold indoors but not to classify the observations into the corresponding mold species present.

## 7. Summary and Conclusions

In the present work, the applications of MOXs-based gas sensors and sensor arrays for environmental monitoring purpose have been overviewed. The environmental pollution is of particular importance in today’s society such that the full attention of national and international environmental institutions as well as researchers are focused on finding solutions to respond to the urgent need for environmental protection. Pollutant gases emissions in some industrial activities cannot be completely avoided despite a wide range of reducing agents. This can disturb and damage human health and the environment. Identifying and controlling the environmental effects of pollutants as a result of the activities of industrial plants is a key element for preventing and reducing industrial pollution and minimizing its impact on humans, which has become mandatory in many countries. In addition, water pollution is constantly intensified by human activities, which can lead to irreversible consequences for humans and even animals. Therefore, useful solutions are offered, as timely measures can prevent the further spread of pollution and even monitor the environment. In this regard, the MOXs gas sensors and sensor arrays, commonly called e-noses, are a very convenient tool for monitoring the environment, and their application involves many degrees of freedom, for example in sampling, training and data processing methods. However, the most important aspect limiting the use of gas sensors and electronic noses for environmental applications is the lack of specific regulations for their standardization. The definition and standardization of the features and performance of e-noses tools and their application methods are prerequisites of their publication.

## Figures and Tables

**Table 1:** The threshold limits for common pollutants established by regulatory bodies such as the European Union and US agencies along with the limit of detection (LoD) values reported in the literature for the best-performing sensors.

Pollutant	US Threshold Limits	EU Threshold Limits	LoD	References
CO_2_	N/A	N/A	150 ppb	[87]
CO	9 ppm	10 ppm	1 ppm	[88]
NO_2_	53 ppb	50 ppb	5 ppb	[89]
O_3_	120 ppb	70 ppb	20 ppb	[90]
SO_2_	75 ppb	130 ppb	38 ppb	[91]

**Table 2:** E-nose application for evaluating selected wastewater parameters.

Analysis Objectives	Sensors Used-Number × Type (Device Manufacturer *); Measurement Protocol *	Obtained Results	Literature Source
Wastewater identification, determination of BOD	12 × CP (Neotronics Scientific Ltd., Chelmsford, England, model D); flowrate 600 mL/min, odor profiles from 1 min	Wastewater classification, correlation of e-nose response with BOD	[92]
Wastewater identification, determination of BOD	12 × CP (Neotronics Scientific Ltd., Chelmsford, England, model D)	Wastewater classification, correlation of e-nose response with BOD	[92]
Determination of COD, TSS and turbidity	12 × MOS (Alpha M.O.S., Toulouse, France, FOX3000); flowrate 150 mL/min, measurement time 60 s	Weak correlation between e-nose response and parameters: COD (R = 0.53) TSS (R = 0.52), turbidity (R = 0.53)	[93]
Wastewater identification	10 × MOS (Airsense Analysentechnik, Schwerin, Germany, Pen-2); 32 × CP (Cyrano Sciences, Pasadena, CA, USA Cyranose 320)	Distinction between odor samples collected from different locations in the treatment plant	[94]
Wastewater identification, odor concentration in the vicinity of the treatment plant	6 × MOS (EOS25), 6 × MOS (EOS28), 6 × MOS (EOS35); measurement 3 min, treatment 12 min	High success rate in identifying odor sources (R = 0.95 for the range of 100 ÷ 150 ou_E_/m^3^), high correlation (R > 0.9) with odor concentrations in the range of 20 ÷ 80 ou_E_/m^3^	[95]
Odor concentration	12 × CP (Neotronics Scientific Ltd., Chelmsford, England, model D); flowrate 600 mL/min, odor profiles from 1 min	Weak correlation between TON and e-nose response in the range of 125 ÷ 781,066 ou_E_/m^3^	[96]
Detection of hazardous pollutants	8 × CP (ProSat, Marconi Applied Technologies, Chelmsford, England, eNOSE 5000); pretreatment 40 s, measurement 1 min, post-treatment 3 min 20 s	Real-time detection of unknown pollutants, illegal and accidental discharges into the sewage network	[97,98,99]
Odor concentration and stability of processes in bioreactor	8 × MOS TGS (Figaro, Tokyo, Japan); odor profiles measurements from more than 370 h of experiment	High correlation between measurements of odor nuisances by e-nose based on MOS sensors and reference method–dynamic olfactometry	[48,100]
Assessment of basic wastewater parameters treated in laboratory sequencing batch reactor with activated sludge	8 × MOS TGS (Figaro, Tokyo, Japan); measurements of one-week experiment–correlation between pollution indicators and e-nose readouts	High correlation (r) between e-nose based on MOS sensors readouts and pollution indicators COD (0.988), TSS (0.938), turbidity (0.940), N-NH_3_ (0.978), N-NO_2_ (0.0.869), N-NO_3_ (0.958), VOC (0.98)	[101]
Classification of malfunction in laboratory sequencing batch reactor with activated sludge	8 × MOS TGS (Figaro, Tokyo, Japan); measurements of 60 days experiment with measurement frequency 1 Hz	The detection accuracy of individual states at level 78.04%	[102]
Application of e-nose for classification of treatment effects at full-scale WWTP	17 × MOS TGS (Figaro, Tokyo, Japan); the total size of multidimensional dataset 185	Very good accuracy for training and testing data by decision tree (i.e., 98% and 97%) and random forest (100%) for classification of treatment effect (stages) at full-scale WWTP	[103,104]
Analysis of water quality parameters and odor concentration of wastewater	32 × MOS sensors with sampling rate of 100 HZ	Recognition rate of sampling points at WWTP at 98.83%, where water parameters and odor concentration were predicted with RMSE less than 0.9476	[105]

* If available; TSS—total suspended solids; TOC—threshold odor number.

## Data Availability

All important information and data are available inside of article.
